# Non-linear association between C-reactive protein levels and length of stay in pediatric appendicitis patients undergoing laparoscopic appendectomy

**DOI:** 10.3389/fped.2024.1464193

**Published:** 2024-12-12

**Authors:** Ming Liu, Ping Yang, Yunpeng Gou

**Affiliations:** Department of Pediatric Surgery, Suining Central Hospital, Suining, Sichuan Province, China

**Keywords:** C-reactive protein, length of stay, appendicitis, laparoscopic appendectomy, children

## Abstract

**Objective:**

To examine the correlation between C-reactive protein (CRP) levels at hospital admission and the length of stay (LOS) in pediatric patients with appendicitis who underwent laparoscopic appendectomy.

**Methods:**

We retrospectively collected the clinical data from pediatric patients diagnosed with acute appendicitis and treated with laparoscopic appendectomy. Multivariate generalized linear regression analyses were performed to determine the independent relationship between CRP and LOS. Smooth curve fitting was constructed to examine the potential non-linear relationship between CRP and LOS. A segmented regression model was used to calculate threshold effects and determine the inflection point.

**Results:**

A total of 815 participants were included in the study. Multiple linear regression analysis indicated that the higher the CRP levels, the longer the LOS. Fully adjusted smooth curve fitting suggested a non-linear relationship between CRP and LOS. A segmented regression showed that the inflection point value of CRP was 34.13 mg/L. A 1 mg/L increase in CRP levels was significantly associated with a 0.013-day increase in length of stay (95% CI: 0.009, 0.018; *P* < 0.001) when CRP levels > 34.13 mg/L. However, there was no significant association between CRP and LOS when CRP levels < 34.13 mg/L (*P* > 0.05).

**Conclusion:**

There was a non-linear association and threshold effect between CRP levels and LOS. CRP levels above 34.13 mg/L were associated with longer LOS in pediatric appendicitis patients. These findings contribute to the understanding of inflammatory markers in recovery dynamics and underscore the necessity for further research to investigate their potential clinical implications.

## 1 Introduction

Acute appendicitis is the most common surgical abdominal emergency in children (1), typically treated with laparoscopic appendectomy (LA) (2–4). C-reactive protein (CRP) is an acute-phase reaction protein predominantly synthesized in the liver in response to acute injury, infection, or other inflammatory stimuli (5). During appendiceal inflammation, the body promotes the release of pro-inflammatory cytokines, which in turn stimulate the expression of CRP, leading to elevated serum CRP levels (6, 7). Moreover, CRP levels are instrumental in monitoring inflammation and infection. Currently, CRP is extensively utilized in diagnosing appendicitis in children and distinguishing complicated appendicitis (8–10).

The duration of postoperative hospital stay serves as a crucial indicator for assessing the recovery of pediatric patients following laparoscopic appendectomy. This metric also reflects resource consumption and healthcare costs (11). However, little research has examined the relationship between CRP levels and length of stay (LOS) in children diagnosed with appendicitis.

Therefore, we aim to examine the correlation between CRP levels at hospital admission and the duration of hospitalization in pediatric patients with appendicitis who underwent laparoscopic appendectomy. This study aims to provide a deeper understanding of the role of CRP in postoperative recovery, contributing to the broader academic discourse on inflammation markers and recovery metrics.

## 2 Materials and methods

### 2.1 Patient selection

We retrospectively collected the clinical data from pediatric patients under the age of 18 diagnosed with acute appendicitis and treated with laparoscopic appendectomy at the pediatric surgery department of Suining Central Hospital between January 2021 and December 2022.

The exclusion criteria for this study are as follows: 1. negative appendectomy (normal appendix); 2. periappendiceal abscess (initially treated with antibiotics or drainage, followed by laparoscopic surgery); 3. presence of other specific infectious diseases, such as lung infection; 5. during intraoperative exploration, various pathologies were identified including Merkel diverticulum, volvulus, oophoritis, adnexal torsion, torsion of ovarian cyst pedicle, rupture of corpus luteum cyst, and other causes of acute abdomen; 6. prior use of antibiotics before admission; 7. presence of concomitant blood system diseases, endocrine system diseases, or other severe medical conditions.

Research approval was obtained from the Ethics Committee of Suining Central Hospital, and due to its retrospective nature, the study did not require informed consent.

### 2.2 Variables

The primary outcome variable in this study was the LOS, and the major exposure factor was CRP. CRP levels were measured at hospital admission, followed by a prompt assessment for surgical intervention. Additionally, CRP elevation was defined as serum CRP levels above 10 mg/L (12, 13). We treated CRP as a continuous independent variable and a categorical variable (divided it into CRP ≤ 10 mg/L and CRP > 10 mg/L) to ensure a robust analysis of its potential effects.

In addition, the covariates were chosen based on established associations in relevant literature (9, 14–17) and clinical significance. Information such as gender, age, height, weight, body mass index (BMI), onset time (from symptom onset to hospital admission), history of fever (>37.5℃ before admission), symptoms of emesis, diarrhea, peritonitis, body temperature on hospital admission, laboratory indexes upon admission, neutrophil-to-lymphocyte ratio (NLR), presence of appendiceal fecalith, drainage tube placement, and classification of appendicitis was collected. Acute appendicitis is classified into simple appendicitis (congestive appendicitis and suppurative appendicitis), gangrenous appendicitis, and perforated appendicitis, depending on the surgical and pathological findings.

### 2.3 Statistical analysis

All continuous variables were analyzed using the Mann-Whitney U test or Kruskal Wallis test and were presented as median (interquartile range, IQR) because of skewed distribution. Categorical variables were presented as frequencies (percentages) and analyzed using the chi-square test. A box plot was employed to visually represent the distribution of LOS across different types of appendicitis.

Multivariate generalized linear regression analyses were performed to determine the independent relationship between CRP and length of stay. The crude model was adjusted for no covariate. Model I was adjusted for gender, age, and classification of appendicitis. Model II was adjusted for gender, age, classification of appendicitis, BMI, onset time, peritonitis, temperature, WBC, neutrophil ratio, NLR, hemoglobin, platelet, albumin, operative time, appendiceal fecalith and drainage tube.

We additionally performed subgroup analyses stratified by gender, peritonitis, appendiceal fecalith, drainage tube, and classification of appendicitis. In order to examine the potential non-linear relationship between CRP and length of stay, a fully adjusted model with smooth curve fitting was constructed. A segmented regression model was used to calculate threshold effects and determine the inflection point.

All statistical analyses were completed by using Empower Stats (version 4.2; http://www.empowerstats.com) and R software (version 4.4.0; https://www.R-project.org). A two-tailed *P*-value < 0.05 was considered to indicate statistical significance.

## 3 Results

### 3.1 Characteristics of participants

**Figure 1:**
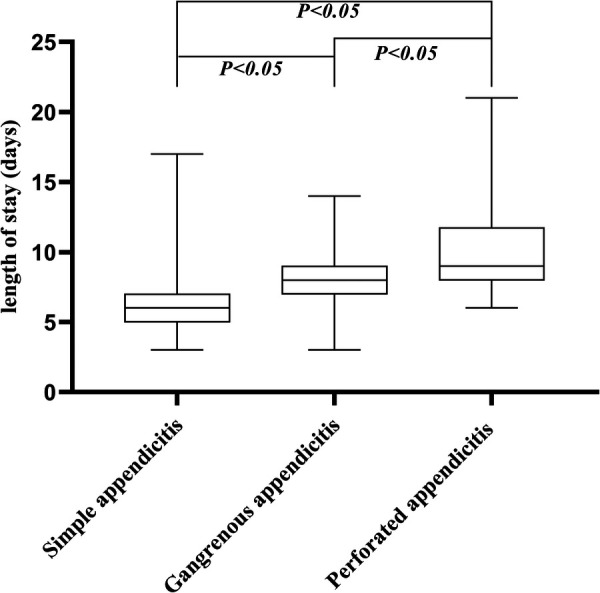
Length of stay in days for pediatric patients with different types of appendicitis. Boxes indicate the median and interquartile range (IQR); whiskers indicate the highest and lowest values.

A total of 815 participants were included in the study. Table 1 shows the characteristics of the study population according to the categories of CRP levels. There were 528 patients (64.79%) with low CRP levels (≤10 mg/L) and 287 patients (35.21%) with elevated CRP levels (>10 mg/L). Of all the participants, 35.95% were females, and 64.05% were males. The median (interquartile range) of length of stay was 6.00 (5.00–7.00) days. For the patients with elevated CRP levels (>10 mg/L), the length of stay tended to be longer compared to the patients with low CRP levels (≤10 mg/L). The boxplot (Figure 1) shows significant differences in LOS among pediatric patients with simple, gangrenous, and perforated appendicitis (*P* < 0.05). Patients with perforated appendicitis had the longest median LOS, followed by those with gangrenous and simple appendicitis. The variability in LOS was greatest in the perforated appendicitis group, as indicated by the larger IQR and whiskers.

**Table 1:** Baseline characteristics of participants.

	Total	CRP group (mg/L)		*P*-value
		CRP ≤ 10	CRP > 10	
Participants	815	528	287	
Length of stay (days)	6.00 (5.00–7.00)	6.00 (5.00–7.00)	7.00 (6.00–9.00)	<0.001
Gender				0.211
Female	293 (35.95%)	198 (37.50%)	95 (33.10%)	
Male	522 (64.05%)	330 (62.50%)	192 (66.90%)	
Age (years)	8.00 (6.00–11.00)	9.00 (6.00–11.00)	7.00 (4.00–10.00)	<0.001
Height (cm)	133.00 (117.00–148.00)	136.00 (123.00–150.25)	126.00 (109.50–140.50)	<0.001
Weight (kg)	28.80 (21.00–41.00)	31.45 (23.08–43.52)	25.00 (18.20–36.70)	<0.001
BMI（Kg/m^2^）	16.37 (14.88–19.09)	16.50 (14.94–19.34)	16.00 (14.70–18.48)	0.033
Onset time (hours)	24.00 (12.00–48.00)	19.00 (8.00–24.00)	24.00 (24.00–48.00)	<0.001
History of fever				<0.001
No	553 (67.85%)	435 (82.39%)	118 (41.11%)	
Yes	262 (32.15%)	93 (17.61%)	169 (58.89%)	
Emesis				<0.001
No	405 (49.69%)	289 (54.73%)	116 (40.42%)	
Yes	410 (50.31%)	239 (45.27%)	171 (59.58%)	
Diarrhea				0.362
No	756 (92.76%)	493 (93.37%)	263 (91.64%)	
Yes	59 (7.24%)	35 (6.63%)	24 (8.36%)	
Peritonitis				<0.001
No	270 (33.13%)	208 (39.39%)	62 (21.60%)	
Yes	545 (66.87%)	320 (60.61%)	225 (78.40%)	
Temperature (℃)	36.70 (36.50–37.00)	36.60 (36.40–36.90)	36.80 (36.50–37.35)	<0.001
WBC (×10^9^/L)	12.60 (8.30–16.70)	11.00 (7.70–15.72)	14.40 (10.80–18.60)	<0.001
Neutrophil ratio (%)	78.10 (64.35–85.10)	74.50 (58.03–84.20)	80.60 (73.15–85.90)	<0.001
Lymphocyte ratio (%)	14.60 (8.30–25.80)	17.00 (9.10–31.72)	11.90 (7.40–17.50)	<0.001
NLR	5.38 (2.54–10.43)	4.42 (1.85–9.35)	6.80 (4.29–11.63)	<0.001
RBC (×10^9^/L)	4.58 (4.32–4.84)	4.60 (4.38–4.85)	4.53 (4.23–4.80)	0.002
Hemoglobin (g/L)	127.00 (120.00–133.00)	128.00 (122.00–134.00)	123.00 (116.00–131.00)	<0.001
Platelet (×10^9^/L)	285.00 (235.50–337.00)	285.00 (238.75–331.25)	284.00 (231.50–341.50)	0.681
ALT (U/L)	13.20 (10.75–18.00)	13.95 (11.00–19.00)	12.90 (10.00–16.40)	<0.001
AST (U/L)	26.20 (22.00–31.00)	26.40 (22.00–31.33)	26.00 (21.10–30.95)	0.167
Direct bilirubin (umol/L)	2.30 (1.60–3.40)	2.20 (1.50–3.00)	2.80 (1.90–4.45)	<0.001
Indirect bilirubin (umol/L)	5.50 (3.50–8.00)	4.90 (3.20–7.20)	6.90 (4.35–9.45)	<0.001
Albumin (g/L)	46.30 (44.10–48.80)	47.05 (45.10–49.32)	44.80 (41.80–47.25)	<0.001
Operative time (minutes)	40.00 (35.00–55.00)	40.00 (30.00–50.00)	55.00 (40.00–70.00)	<0.001
Appendiceal fecalith				<0.001
No	579 (71.04%)	424 (80.30%)	155 (54.01%)	
Yes	236 (28.96%)	104 (19.70%)	132 (45.99%)	
Drainage tube				<0.001
No	593 (72.76%)	460 (87.12%)	133 (46.34%)	
Yes	222 (27.24%)	68 (12.88%)	154 (53.66%)	
Classification of appendicitis				<0.001
Simple appendicitis	657 (80.61%)	499 (94.51%)	158 (50.05%)	
Gangrenous appendicitis	62 (7.61%)	15 (2.84%)	47 (16.38%)	
Perforated appendicitis	96 (11.78%)	14 (2.65%)	82 (28.57%)	

CRP, c-reactive protein; BMI, body mass index; WBC, white cell count; NLR, neutrophil-to-lymphocyte ratio; RBC, red blood cell; ALT, alanine aminotransferase; AST, aspartate aminotransferase.

### 3.2 Multivariate linear regression analysis

Table 2 showed a positive correlation between CRP (per 10 mg/L) and length of stay in the non-adjusted model [*β* (95% CI) = 0.263 (0.233, 0.294)], minor adjusted model [0.160 (0.126, 0.195)], and full adjusted model [0.087 (0.053, 0.122)]. Multiple linear regression analysis indicated that the higher the CRP levels, the longer the length of stay. When CRP was classified as a categorical variable, participants with higher CRP levels (>10 mg/L) had a significantly longer LOS compared to participants with lower CRP levels (≤10 mg/L) in the crude model [1.599 (1.313, 1.884)], and model I [0.612 (0.317, 0.908)].

**Table 2:** Association between CRP and length of stay.

	Crude model *β* (95% CI), *P*	Model I *β* (95% CI), *P*	Model II *β* (95% CI), *P*
Continuous CRP per 10 mg/L	0.263 (0.233, 0.294) < 0.001	0.160 (0.126, 0.195) < 0.001	0.087 (0.053, 0.122) < 0.001
CRP group			
≤10 mg/L	Reference	Reference	Reference
>10 mg/L	1.599 (1.313, 1.884) < 0.001	0.612 (0.317, 0.908) < 0.001	0.075 (−0.205, 0.356) 0.598

Crude model: non-adjusted model.

Model I: adjusted for: gender, age and classification of appendicitis.

Model II: adjusted for: gender, age, classification of appendicitis, BMI, onset time, peritonitis, temperature, WBC, neutrophil ratio, NLR, hemoglobin, platelet, albumin, operative time, appendiceal fecalith and drainage tube.

CRP, c-reactive protein; BMI, body mass index; WBC, white cell count; NLR, neutrophil-to-lymphocyte ratio.

### 3.3 Subgroup analyses

We further performed the stratified analysis by gender, peritonitis, appendiceal fecalith, drainage tube, and classification of appendicitis in all models (Table 3). As can be seen in model II, the positive correlation between CRP and LOS was significant in each subgroup of gender and appendiceal fecalith. However, in the classification of appendicitis subgroups, the relationship between CRP and LOS was significant in simple appendicitis, but not significant in gangrenous appendicitis and perforated appendicitis. Additionally, there was a significant positive correlation between CRP and LOS among patients with peritonitis and a drainage tube.

**Table 3:** Subgroup analyses of the association between CRP and length of stay.

	Crude model *β* (95% CI), *P*	Model I *β* (95% CI), *P*	Model II *β* (95% CI), *P*
CRP per 10 mg/L	0.263 (0.233, 0.294) < 0.001	0.160 (0.126, 0.195) < 0.001	0.087 (0.053, 0.122) < 0.001
Gender			
Female	0.270 (0.217, 0.322) < 0.001	0.125 (0.058, 0.192) < 0.001	0.088 (0.019, 0.157) 0.013
Male	0.258 (0.221, 0.296) < 0.001	0.169 (0.128, 0.210) < 0.001	0.087 (0.046, 0.128) < 0.001
Peritonitis			
No	0.116 (0.029, 0.202) 0.009	0.070 (−0.022, 0.162) 0.138	0.042 (−0.054, 0.138) 0.393
Yes	0.273 (0.240, 0.306) < 0.001	0.172 (0.134, 0.210) < 0.001	0.093 (0.057, 0.130) < 0.001
Appendiceal fecalith			
No	0.194 (0.148, 0.240) < 0.001	0.157 (0.107, 0.207) < 0.001	0.082 (0.029, 0.135) 0.002
Yes	0.224 (0.174, 0.273) < 0.001	0.144 (0.089, 0.198) < 0.001	0.078 (0.024, 0.132) 0.005
Drainage tube			
No	0.094 (0.038, 0.149) 0.001	0.068 (0.009, 0.126) 0.023	0.059 (−0.002, 0.121) 0.060
Yes	0.164 (0.116, 0.212) < 0.001	0.130 (0.079, 0.181) < 0.001	0.081 (0.029, 0.134) 0.003
Classification of appendicitis			
Simple appendicitis	0.159 (0.114, 0.205) < 0.001	0.160 (0.114, 0.206) < 0.001	0.083 (0.037, 0.128) < 0.001
Gangrenous appendicitis	0.197 (0.100, 0.294) < 0.001	0.149 (0.047, 0.250) 0.006	0.039 (−0.084, 0.161) 0.539
Perforated appendicitis	0.145 (0.063, 0.228) < 0.001	0.141 (0.057, 0.226) 0.002	0.083 (−0.003, 0.170) 0.063

Crude model: non-adjusted model.

Model I: adjusted for: gender, age and classification of appendicitis.

Model II: adjusted for: gender, age, classification of appendicitis, BMI, onset time, peritonitis, temperature, WBC, neutrophil ratio, NLR, hemoglobin, platelet, albumin, operative time, appendiceal fecalith and drainage tube.

CRP, c-reactive protein; BMI, body mass index; WBC, white cell count; NLR, neutrophil-to-lymphocyte ratio.

### 3.4 Non-linear analysis

**Figure 2:**
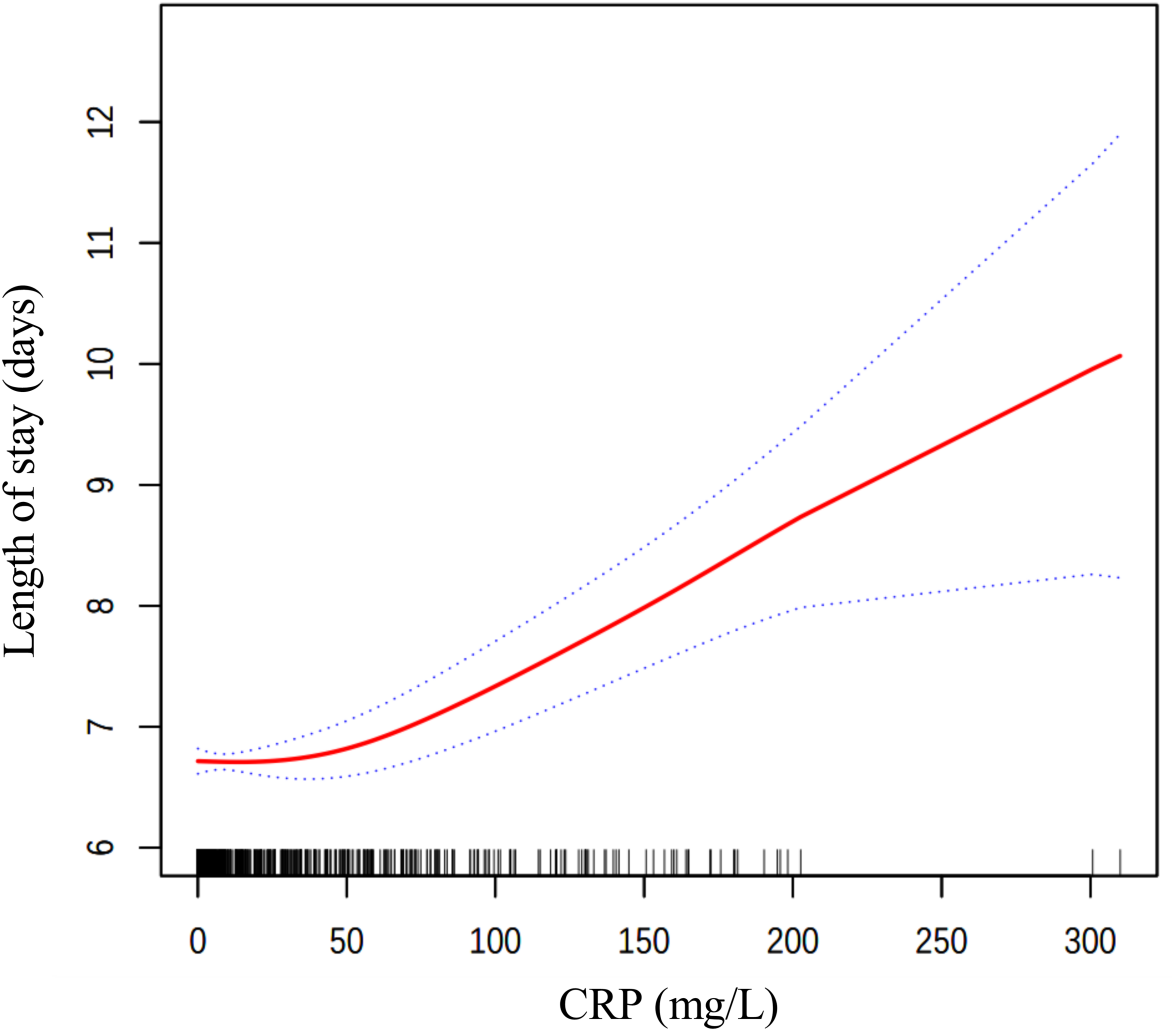
The relationship between CRP levels and length of stay. Solid line in the middle represents smooth curve fitting between variables. The dotted line on both sides indicates a 95% confidence interval (CI). Black and white stripes at the bottom show density based on CRP levels. All models were adjusted for gender, age, classification of appendicitis, BMI, onset time, peritonitis, temperature, WBC, neutrophil ratio, NLR, hemoglobin, platelet, albumin, operative time, appendiceal fecalith and drainage tube. CRP, c-reactive protein; BMI, body mass index; WBC, white cell count; NLR, neutrophil-to-lymphocyte ratio.

As shown in Figure 2, fully adjusted smooth curve fitting suggested a non-linear relationship between CRP and LOS. A segmented regression showed that the inflection point value of CRP was 34.13 mg/L (Table 4). A 1 mg/L increase in CRP levels was significantly associated with a 0.013-day increase in length of stay (95% CI: 0.009, 0.018; *P* < 0.001) when CRP levels > 34.13 mg/L. However, there was no significant association between CRP and LOS when CRP levels < 34.13 mg/L (*P* > 0.05, log-likelihood ratio test = 0.002).

**Table 4:** Threshold effect analysis for the relationship between CRP and length of stay.

	Length of stay (days)	
	Adjusted *β* (95% CI)	*P*-value
Model I		
the standard linear mode	0.009 (0.005, 0.012)	<0.001
Model II		
Inflection point	34.13	
CRP < 34.13 mg/L	−0.007 (−0.018, 0.004)	0.188
CPR > 34.13 mg/L	0.013 (0.009, 0.018)	<0.001
Log likelihood ratio test	0.002	

Adjusted for: gender, age, classification of appendicitis, BMI, onset time, peritonitis, temperature, WBC, neutrophil ratio, NLR, hemoglobin, platelet, albumin, operative time, appendiceal fecalith and drainage tube.

CRP, c-reactive protein; BMI, body mass index; WBC, white cell count; NLR, neutrophil-to-lymphocyte ratio.

## 4 Discussion

This study contributes to the existing literature by quantifying the non-linear relationship between preoperative CRP levels and the length of hospital stay in pediatric patients undergoing laparoscopic appendectomy. Our findings enhance the academic understanding of how inflammatory markers like CRP may influence recovery trajectories and establish a foundation for future research on CRP's predictive value in postoperative care. Specifically, we observed an inflection point of 34.13 mg/L for CRP; on the right side of the inflection point, higher CRP levels are significantly associated with prolonged length of hospital stay, with each one mg/L increase in CRP corresponding to a 0.013-day increase in length of stay. Conversely, on the left side of the inflection point, the relationship between CRP levels and length of hospital stay is not statistically significant. This threshold indicates that CRP levels exceeding 34.13 mg/L are linked to prolonged hospitalization. Although this finding enhances the academic understanding of inflammatory markers, its direct application in clinical management necessitates further validation through prospective studies.

The duration of postoperative hospitalization following laparoscopic appendectomy is subject to variability based on the public health insurance policies of the state and the individual surgeon's practices. Our study observed a mean hospital stay of 6.00 (5.00–7.00) days, exceeding the durations reported in previous literature (3, 4, 18–20), potentially influenced by the following factors: The study group comprises individuals of a younger age, making clinical diagnosis challenging and leading to rapid disease progression. They also lack cooperation with medical work and require more post-surgery recovery attention. Meanwhile, our hospital is a tertiary general hospital where many children are referred from local or nearby hospitals, causing delays in diagnosis and treatment. Additionally, all discharged patients in this study exhibited no clinical symptoms, including fever and abdominal pain, and demonstrated a return to normal levels of inflammation indicators such as white blood cell count and C-reactive protein. Also, patients could resume a regular diet and normal intestinal motility. Thus, the length of stay for these patients can be considered indicative of complete disease control.

CRP, identified as a serum acute-phase reactant in 1930, exhibits a half-life of 19 h and is primarily synthesized in the liver. Its release is triggered by pro-inflammatory cytokines, notably interleukin-6 and tumor necrosis factor-α (5). CRP is capable of activating the complement pathway and enhancing the clearance of foreign and damaged cells (21). CRP levels are frequently elevated in various inflammatory states and exhibit rapid fluctuations, typically rising shortly after the onset of inflammation and returning to baseline levels within 1 week (22). Consequently, CRP serves as a valuable biomarker for monitoring treatment efficacy and disease progression.

Past research (23–25) has demonstrated that CRP serves as a non-specific marker of inflammation, with increased plasma CRP levels indicating infection and aiding in the clinical assessment of appendicitis. Zouari et al. (26) underscore the significance of CRP values exceeding 10 mg/L as robust indicators of acute appendicitis in children below the age of 6. Furthermore, research (27, 28) has demonstrated that CRP levels can aid in the identification of complicated appendicitis, with particularly elevated levels observed in cases of perforated appendicitis (29–33). Recent research (34) indicates that serum CRP levels upon admission can serve as a prognostic indicator for acute appendicitis in pediatric patients. Those presenting with CRP levels exceeding 3 mg/dl are at an elevated risk for peritonitis and should receive heightened monitoring and prompt, aggressive treatment.

The findings of our study indicate a significant positive correlation between CRP and LOS in patients with appendicitis. This relationship may be attributed to CRP as a marker for the inflammatory response, with elevated levels reflecting the severity of appendicitis and the degree of infection-related inflammation. Higher CRP levels may suggest a more severe disease state, potentially necessitating a more extended post-operative recovery period.

Research (35) has demonstrated a correlation between elevated preoperative CRP levels and extended hospitalization following appendicitis, with a separate study (16) confirming that CRP levels exceeding 150 mg/L serve as an independent prognostic indicator for prolonged hospital stays in appendicitis patients. Both studies demonstrated a relationship between CRP and LOS from varying viewpoints. However, both studies treated CRP as a categorical variable and simply identified it as a risk factor for prolonged hospitalization following appendicitis without providing a quantitative analysis of this association. Furthermore, the first study focused on adults, whereas the second study exclusively examined children with appendicitis who had not received surgical intervention. Nevertheless, our research enhances existing studies by examining CRP as a categorical and continuous variable. This study quantifies the correlation between CRP levels and LOS in pediatric patients, identifies a threshold effect and inflection point, and establishes the optimal CRP threshold value. The investigation also sheds light on the relationship between CRP and LOS in various scenarios, offering a fresh perspective on the role of CRP in pediatric appendicitis. In contrast, a separate investigation (36) revealed no significant correlation between preoperative CRP levels and LOS following appendicitis surgery. This study differed from ours in several vital aspects, including a smaller sample size, a restricted study population limited to uncomplicated appendicitis cases, and the analysis of CRP solely as a categorical variable.

The findings of this study indicate that while multiple regression analysis revealed a positive correlation between CRP levels and LOS, further stratified analysis revealed significant variations in this association among subgroups categorized by peritonitis, drainage tube presence, and pathological types of appendicitis. Specifically, a significant positive correlation was observed in cases of peritonitis, presence of drainage tube, and simple appendicitis. Conversely, no significant correlation was observed in subgroups without peritonitis, without a drainage tube, or in cases of gangrenous or perforated appendicitis. This disparity indicates that the correlation between CRP and LOS could potentially be influenced by factors such as peritonitis, drainage tube placement, and the specific pathologic characteristics of appendicitis. This discovery underscores the importance of taking into account the regulatory impact of peritonitis, drainage tube placement, and pathologic subtypes of appendicitis when investigating the determinants of hospitalization duration following laparoscopic appendectomy in pediatric patients, and warrants further investigation into the underlying mechanisms. Hence, it is recommended that tailored hospitalization time management protocols be developed for various types of peritonitis, drainage tubes, and pathological appendicitis in both clinical practice and public health strategy development to optimize the utilization of medical resources and reduce healthcare expenditures. Subsequent research endeavors should investigate the underlying factors contributing to these discrepancies to corroborate and enhance our conclusions.

However, the study was subject to certain limitations. Firstly, the retrospective design introduced the potential for information and selection bias. Secondly, the sample consisted of a higher proportion of males than females, impacting the external validity of the findings. Future research should aim to address this gender imbalance. In addition, the study did not fully control for potential confounding factors, such as interleukin 6 and serum procalcitonin.

In conclusion, this study demonstrated a non-linear association and threshold effect between CRP and length of stay in pediatric patients with appendicitis who underwent laparoscopic appendectomy. The inflection point for CRP was determined to be 34.13 mg/L. Above this threshold, a significant positive correlation existed between CRP levels and length of stay. This finding highlights a potential avenue for future clinical research to investigate the implications of CRP levels on patient recovery, while its immediate clinical application necessitates further exploration.

## Data Availability

The data analyzed in this study is subject to the following licenses/restrictions: The datasets generated during and/or analyzed during the current study are not publicly available due to individual privacy, but are available from the corresponding author on reasonable request. Requests to access these datasets should be directed to Ping Yang 13320245381@163.com.
